# Optimal Control Strategy Design Based on Dynamic Programming for a Dual-Motor Coupling-Propulsion System

**DOI:** 10.1155/2014/958239

**Published:** 2014-07-23

**Authors:** Shuo Zhang, Chengning Zhang, Guangwei Han, Qinghui Wang

**Affiliations:** National Engineering Laboratory for Electric Vehicles, School of Mechanical Engineering, Beijing Institute of Technology, Beijing 100081, China

## Abstract

A dual-motor coupling-propulsion electric bus (DMCPEB) is modeled, and its optimal control strategy is studied in this paper. The necessary dynamic features of energy loss for subsystems is modeled. Dynamic programming (DP) technique is applied to find the optimal control strategy including upshift threshold, downshift threshold, and power split ratio between the main motor and auxiliary motor. Improved control rules are extracted from the DP-based control solution, forming near-optimal control strategies. Simulation results demonstrate that a significant improvement in reducing energy loss due to the dual-motor coupling-propulsion system (DMCPS) running is realized without increasing the frequency of the mode switch.

## 1. Introduction

The application of battery electric vehicle in public transport field is a good way to improve the increasing air pollution and shortage of oil resources problems. Developing the electric bus has significant meanings for energy saving, emission reduction, and the electric vehicle (EV) industry development. The control of high-power drive system is one of the key technologies for electric bus. Dual-motor drive coupled by planetary gear is an effective way to realize the high-power drive system. Owing to the dual-power source nature, the control strategy of DMCPEB is typically more complicated than that of traditional engine based vehicle. Therefore, system-level vehicle simulation methodology is often applied to implement accurate sizing and matching studies and to develop effective energy control method, before the final design and physical prototyping.

The power control strategy for electric vehicle can be roughly classified into three categories (see [1, 2]). The first type employs heuristic control techniques such as control rules/fuzzy logic/neural networks for estimation and control algorithm development (see [3, 4]). The second approach is based on static optimization methods (see [5, 6]). The third type of EV control algorithms considers the dynamic nature of the system when performing the optimization (see [7–9]). In addition, the optimization is with respect to a time horizon, rather than for an instant in time. In general, power split algorithms resulting from dynamic optimization approaches are more accurate under transient conditions but are computationally more intensive.

In this paper, dynamic programming (DP) technique is applied to solve the optimal control strategy problem of a DMCPEB. The optimal control strategy solution over a driving cycle is obtained by minimizing a defined cost function. Two cases are solved: an energy-loss-only case and an energy-loss/shifting-frequency case. The comparison of these two cases provides insight into the change needed when the additional objective of riding comfort is included. However, the DP control actions are not implementable due to their preview nature and heavy computational requirement. They are, on the other hand, a good design tool to analyze, assess, and adjust other control strategies. After studying the behavior of the dynamic programming solution carefully, we extract implementable rules. These rules are used to improve a simple, intuition-based algorithm. It was found that the performance of the rule-based algorithm can be improved significantly.

The paper is organized as follows. In Section 2, the dual-motor coupling-propulsion electric bus model is described, followed by an explanation of the preliminary rule-based control strategy. The dynamic optimization problem and the DP procedure are introduced in Section 3. The optimal results for the energy-loss-only and energy-loss/shifting-frequency optimization cases are given in Section 4.  Section 5 describes the design of improved rule-based control strategies. Finally, conclusions are presented in Section 6.

## 2. DMCPEB Configuration and Preliminary Rule-Based Control Strategy

### 2.1. DMCPEB Configuration and Modeling

**Figure 1:**
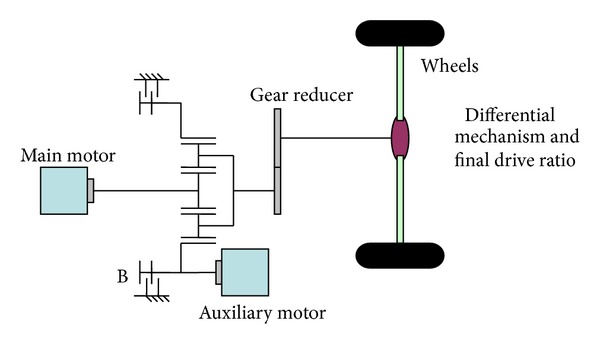
The dual-motors coupling-propulsion electric bus modeling configuration.

The target vehicle is a conventional bus whose engine and part transmission were replaced by a DMCPS developed by Beijing Institute of Technology [10]. The schematic of the vehicle is given in Figure 1. The power source was the main motor and auxiliary motor and the two powers coupled together through a planetary gear train. The main motor is connected to the sun gear while the auxiliary motor is connected to the ring gear. The coupled power output through planet carrier and the planet carrier was linked to the wheels by transmission system. B stands for a wet clutch which can realize mode switch by locking the ring gear smoothly. Important parameters of the vehicle and DMCPS are given in Table 1.

### 2.2. Preliminary Rule-Based Control Strategy

Compared with HEV, BEV's power management strategy seems much simpler, as most of BEVs only have one driving motor which means that the output power of motor is directly determined by the driver's power requirement. For two-motor coupled driving system, there are four possible operating modes: one-motor driving, two-motor driving, one-motor regenerative braking, and two-motor regenerative braking. In order to reduce the energy loss, the power management controller has to decide which operating mode to use and determine the proper power split between the two power sources while meeting the driver's demand. When the system is working in two-motor condition, the situation can be classified into torque coupling and speed coupling according to the structure of the driving system. For torque coupling driving system, the power split of power sources can be realized by determining the torque split, while, for the speed coupling driving system, the power split of power sources can be realized by determining the speed split between the two sources. The simple rule-based power management strategy was developed on the basis of engineering intuition and simple analysis of vehicle's driving characteristics and vehicle's dynamic requirements [11], which is a very popular design approach in electric vehicle. According to the vehicle status, the operation of the controller is determined by one of the two control modes: mode switch control and power split control. The basic logic of each control rule is described below.

*Mode Switch Control*. Based on the working property of the driving system and the efficiency map of the motor, if the vehicle speed exceeds *v*_*p*+_ or is below *v*_*p*−_, the mode switch control will be applied to determine whether the auxiliary motor works or not, as shown in (2). The relationship of *v*_*p*−_ and *v*_*p*+_ can be expressed as follows:

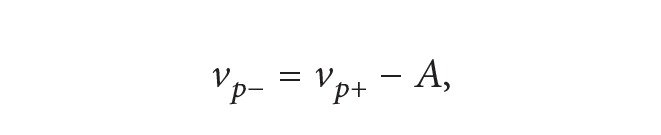
(1)

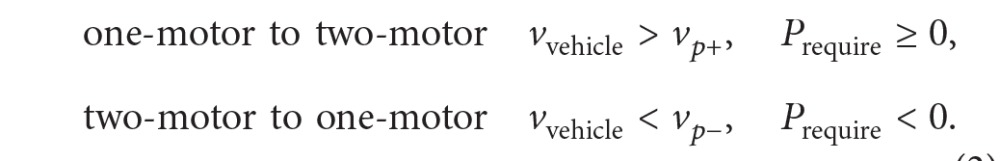
(2)


The speed discrepancy *A* is to avoid continual mode switch which will influence the vehicle ride comfort.

*Power Split Control*. As this vehicle's driving system is speed coupling mode, the power split ratio is proportional to the speed ratio. There are two situations in terms of the power split control. In one-motor working situation, the driving system does not need power split control. The main motor will provide all the needed power, according to the vehicle speed and the pedal motion. In two-motor working situation, considering the motor's efficiency property the main motor will be working on the fixed relative high speed point *N*_main_ and the auxiliary motor speed will change according to the vehicle speed requirements. The output torque of motors will change simultaneously according to the pedal motion. The detailed information of the speed split can be expressed as follows:

(3) $$\begin{array}{c} \begin{array}{c} N_{s}=\frac{S_{\text{vehicle}}\times 60\times \left(1+K\right)}{3.6\times R_{\text{tire}}\times 2\pi \times i_{0}\times i_{2}}\\ Nr=0, \end{array}\;\text{one-motor}\;\;\text{mode},\;\\ \begin{array}{c} N_{s}=N_{\text{main}},\\ Nr=\frac{S_{\text{vehicle}}\times 60\times \left(1+K\right)}{3.6\times R_{\text{tire}}\times 2\pi \times i_{0}\times i_{2}\times K}-\frac{N_{s}}{K} \end{array}\text{two-motor}\;\;\text{mode}, \end{array}$$

where *N*_*s*_ is the speed of main motor, which is connected to the sun gear directly, and *N*_*r*_ is the speed of auxiliary motor, which is connected to the ring gear directly. *K* can be got by *K* = *Z*_*r*_/*Z*_*s*_, *Z*_*r*_ and *Z*_*s*_ is the number of tooth for ring gear and sun gear.

## 3. Dynamic Optimization Problem

Compared with rule-based algorithms, the dynamic optimization approach can find the best control strategy relying on a dynamic model (see [12, 13]). Given a driving cycle, the DP-based algorithm can obtain the optimal operating strategy minimizing the system's energy loss subject to the diverse constraints. A numerical-based DP approach is adopted in this paper to solve this finite horizon dynamic optimization problem.

### 3.1. Problem Formulation

In the discrete-time format, a model of the battery electric vehicle can be expressed as

(4) $$\begin{array}{c} x\left(k+1\right)=f\left(x\left(k\right),u\left(k\right)\right), \end{array}$$

where *u*(*k*) is the vector of control variables such as shifting command of the driving system and desired speed ratio increments of the auxiliary motor. *x*(*k*) is the state vector of the system such as the working mode of the system (one-motor mode or two-motor mode) and the speed ratio of the motors. The sampling time for the control problem is selected to be one second. The optimization goal is to find the control input *u*(*k*) to minimize a cost function, which consists of the weighted sum of energy loss and the frequency of the mode change. The cost function to be minimized has the following form:

(5) J=∑k=0N−1L(x(k),u(k))=∑k=0N−1Lm(k)+La(k)+Lc(k)+α×|Shift(k)|,

where *N* is the duration of the driving cycle and *L* is the instantaneous cost including main-motor energy loss *L*_*m*_(*k*), auxiliary-motor loss *L*_*a*_(*k*), power-coupling gear-box loss *L*_*c*_(*k*), and mode change cost *α* × |Shift(*k*)|. For an energy-only problem, the weighting factor *α* is set to be zero. The case *α* > 0 represents a comprehensive problem which considered the energy loss and the number of mode changes. During the optimization, it is necessary to impose the following inequality constraints to ensure safe/reasonable operation of the main motor and auxiliary motor:

(6) $$\begin{array}{c} T_{s\_\min}\left(N_{s}\left(k\right)\right)\leq T_{s}\left(N_{s}\left(k\right)\right)\leq T_{s\_\max}\left(N_{s}\left(k\right)\right),\\ N_{s\_\min}\leq N_{s}\left(k\right)\leq N_{s\_\max},\\ T_{r\_\min}\left(N_{r}\left(k\right)\right)\leq T_{r}\left(N_{r}\left(k\right)\right)\leq T_{r\_\max}\left(N_{r}\left(k\right)\right),\\ {\;N}_{r\_\min}\leq N_{r}\left(k\right)\leq N_{r\_\max}, \end{array}$$

where *T*_*s*_ is the output torque of the main motor and *T*_*r*_ is the output torque of the auxiliary motor. In addition, to satisfy the systems properties, besides this basic constraints, other constraints are needed:

(7) $$\begin{array}{c} N_{s}\left(k\right)\times N_{r}\left(k\right)\geq 0, \end{array}$$

this constrain is to avoid the power cycling which can increase power loss greatly and is undesirable in reality. Another constrain is that when *N*_*s*_ = 0, the speed of *N*_*r*_ should also be zero. This is because our current system only has one wet clutch and it is fixed with the ring gear. This means that when the vehicle is running, the sun gear must be running too.

### 3.2. Model Simplification

The detailed DMCPS and DMCPEB models are not suitable for dynamic optimization due to their high number of states. Thus, a simplified but sufficiently complex vehicle model is developed. This DMCPS is a speed coupling system and can be calssified into two working modes (one-motor working mode and two-motor working mode). As the two aspects are the main influence factors when the DMCPS's parameters are determined, it was decided that only these two state variables needed to be kept: the two motors' speed ratio and DMCPS's working mode. The simplifications of the subsystems motors, vehicle, transmission, battery, and the planetary gear train are described below.

**Figure 2:**
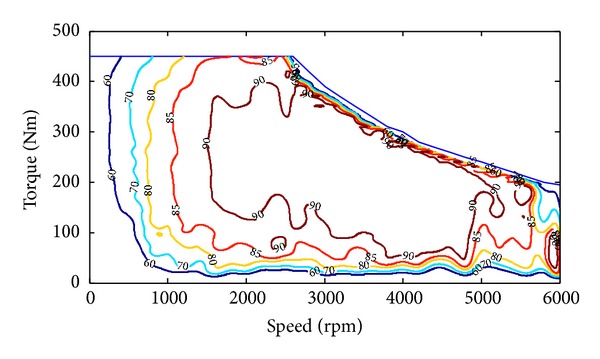
Efficiency map of the electric motor.

*Motors*. The electric motor characteristics are based on the efficiency data obtained from [10] as shown in Figure 2. From Table 1 we can get that though the DMCPS needs two motors, they have the same specifications and they are of the same type. So here we only display one efficiency map of the electric motor. Considering the regenerative braking, here we assume that when the output torque of motor is negative, the efficiency is the same as when the motor output positive torque whose value is the same as the absolute value of the negative torque. The motor efficiency *η*_*m*_ can be expressed as

(8) $$\begin{array}{c} \eta_{m}=f\left(\left|T_{m}\left(N_{m}\right)\right|,N_{m}\right), \end{array}$$

where |*T*_*m*_| is the absolute value of the motor's output torque and *N*_*m*_ is the rotate speed of the motor. When the vehicle is braking in emergency condition, the DMCPS cannot provide enough braking force. Here the braking strategy is determined to be series strategy: when the DMCPS can provide enough brake force, all the brake force will be provided by the DMCPS, and when the needed brake force exceeds the DMCPS's ability, the DMCPS will provide the maximum torque while the extra force will be provided by the friction braking system. The output torque *T*_*m*_(*N*_*m*_) can be expressed as follows:

(9) $$\begin{array}{c} \left|T_{m}\left(N_{m}\right)\right|=\{\begin{array}{cc} \left|T_{m,\text{req}}\right| & \text{if}\;\;\left|T_{m,\max}\left(N_{m}\right)\right|\geq \left|T_{m,\text{req}}\right|\\ \left|T_{m,\max}\left(N_{m}\right)\right| & \text{if}\;\;\left|T_{m,\max}\left(N_{m}\right)\right|<\left|T_{m,\text{req}}\right|, \end{array} \end{array}$$

where *T*_*m*,req_ is the required torque.

*Vehicle*. The vehicle is modeled as a point mass:

(10) Vvehicle(k+1)=Vvehicle(k) +(((Ts(k)×(1+K)×i0×i2−Tb(k))rt−Ff−Fa(Vvehicle(k)))(Mr)−1),

where *T*_*b*_(*k*) is the friction brake force, *i*_0_ is the reduction ratio of the gear reducer, *K* is the property parameter of the planetary gear train, *F*_*f*_ and *F*_*a*_ are the rolling resistance force and the aerodynamic drag force, respectively, *r*_*t*_ is the tire radius, *M*_*r*_ is the effective mass of the vehicle, and *J*_*r*_ is the equivalent moment of inertia of the rotating components in the vehicle. *F*_*f*_, *F*_*a*_, and *M*_*r*_ can be got from the following equations:

(11) $$\begin{array}{c} F_{f}=M_{\text{vehicle}}\times g\times f,\\ F_{a}=\frac{C_{D}Au_{a}}{21.5},\\ M_{r}=M_{\text{vehicle}}+\frac{J_{r}}{r_{t}^{2}}, \end{array}$$

where *M*_vehicle_ is the mass of the vehicle, *g* is the gravity acceleration, *f* is the rolling resistance coefficient, *C*_*D*_ is aerodynamic drag coefficient, *A* is the effective projected area of vehicle, and *u*_*a*_ is the speed of the vehicle.

*Transmission*. The working modes (one-motor working mode and two-motor working mode) are modeled as a discrete-time dynamic system with 1 s time increment

(12) $$\begin{array}{c} m_{x}\left(k+1\right)=\{\begin{array}{cc} 1, & m_{x}\left(k\right)+\text{Shift}\left(k\right)>1\\ 0, & m_{x}\left(k\right)+\text{Shift}\left(k\right)<0\\ m_{x}\left(k\right)+\text{Shift}\left(k\right), & \text{otherwise}, \end{array} \end{array}$$

where state *m*_*x*_ is the main working mode and the control shift to the transmission is constrained to take on the values of −1, 0 and 1, representing downshift, sustain and upshift, respectively.

*The Battery*. The Lithium-Ion Battery is used. A lot of work has been done about estimating the state of charge (SOC) of the battery [14–16], which is very important for HEV and BEV. As this paper mainly focused on the DMCPS and for the BEV the batteries just provide the needed power and cannot be optimized as the needed power is fixed according to the certain drive cycle, here we assume that the battery can always meet the drive cycle's power requirement and no energy loss is coming from the battery.

*The Planetary Gear Train*. Based on the planetary gear train's working property, we can get that different control strategy can also lead to the different energy loss due to the different efficiency, and so the planetary gear train's efficiency model should also be built to calculate the energy loss. As the planetary gear train is a TWO-DOF mechanism, the efficiency *ŋ*_*E*_ can be got from the following formula [17]:

(13) $$\begin{array}{c} {ŋ}_{Ea}=\frac{N_{c}}{N_{s}/\left(1-K\right){ŋ}_{r(s-c)}-N_{r}K/\left(1-K\right){ŋ}_{s(r-c)}}\\ {ŋ}_{Ed}=\frac{Kp{ŋ}_{s(c-r)}N_{r}+{{ŋ}_{r(c-s)}N}_{s}}{\left(1+Kp\right)N_{c}}, \end{array}$$

where *ŋ*_*Ea*_ stands for the efficiency when the vehicle is accelerating, *ŋ*_*Ed*_ stands for the efficiency when the vehicle is decelerating, *ŋ*_*r*(*s*−*c*)_ denotes the efficiency that when the ring gear is fixed, the power is input into the sun gear and output from planet carrier, and *ŋ*_*s*(*r*−*c*)_ denotes the efficiency that when the sun gear is fixed, the power is input into the ring gear and output from planet carrier. *ŋ*_*s*(*c*−*r*)_ denotes the efficiency that when the sun gear is fixed, the power is input into the planet carrier and output from ring gear, and *ŋ*_*r*(*c*−*s*)_ denotes the efficiency that when the ring gear is fixed, the power is input into the planet carrier and output from sun gear.

### 3.3. Dynamic Programming Method

The DP technique is based on Bellman's Principle of Optimality, which states that the optimal policy can be obtained if we first solve a one stage subproblem involving only the last stage and then gradually extend to subproblems involving the last two stages, last three stages,…, and so forth, until the entire problem is solved. In this manner, the overall dynamic optimization problem can be decomposed into a sequence of simpler minimization problems as follows (see [18, 19]).

Step *N* − 1: consider

(14) JN−1∗(x(N−1))   =min⁡u(N−1)⁡[L(x(N−1),u(N−1))+G(x(N))].

Step *k*: for 0 ≤ *k* < *N* − 1,

(15) Jk∗(x(k)) =min⁡u(k)⁡[L(x(k),u(k))+Jk+1∗(x(k+1))],

where *J*_k_*(*x*(*k*)) is the optimal cost-to-go function or optimal value function at state *x*(*k*) starting from time stage *k*. It represents the optimal cost that if at stage *k* the system starts at state *x*(*k*) and follows the optimal control law thereafter until the final stage. The above recursive equation is solved backward to find the optimal control policy. The minimizations are performed subject to the inequality constraints shown in (6) and the equality constraints imposed by the driving cycle.

### 3.4. Numerical Computation

As the DMCPS is a nonlinear system, this DP has to be solved numerically by some approximations. A standard way to solve (15) numerically is to use quantization and interpolation (see [2, 18]). For continuous state space and control space, the state and control values are first discretized into finite grids. At each step of the optimization search, the function *J*_*k*_(*x*(*k*)) is evaluated only at the grid points of the state variables. If the next state *x*(*k* + 1) does not fall exactly on a quantized value, then the values of *J*_k_*(*x*(*k*)) in (15) as well as *G*(*x*(*N*)) in (14) are determined through linear interpolation.

## 4. Dynamic Programing Results

The DP procedure described above produces an optimal, time-varying, state-feedback control law. In the following, two cases are presented: energy-loss-only problem and energy-loss/ mode-change problem.

### 4.1. Energy-Loss Optimization Results

**Figure 3:**
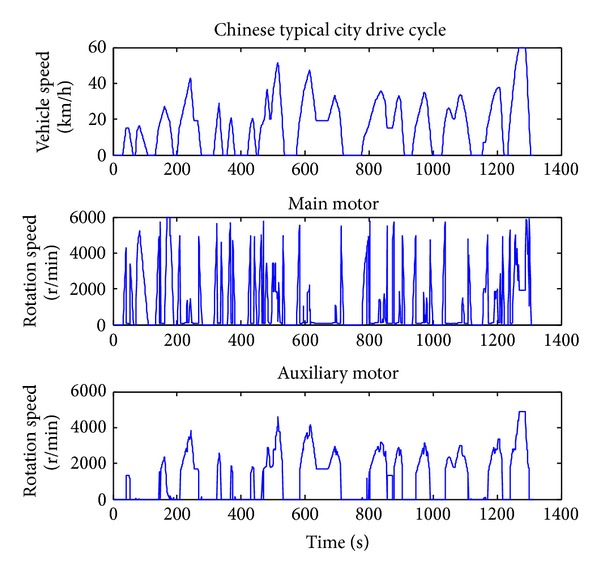
The rotating speed of the two motors.

**Figure 4:**
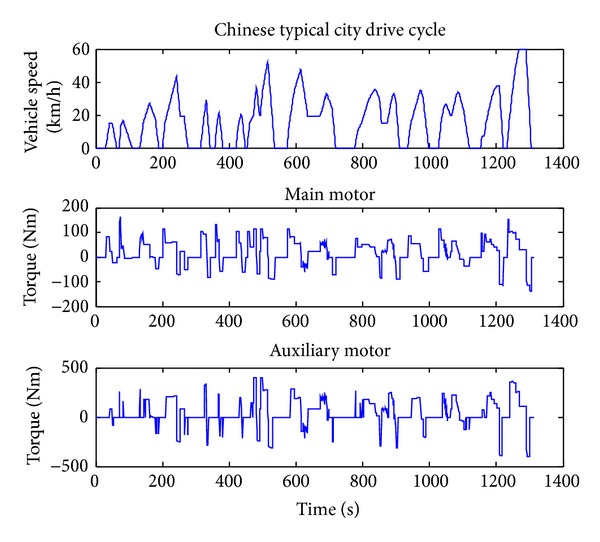
The output torque of the two motors.

**Figure 5:**
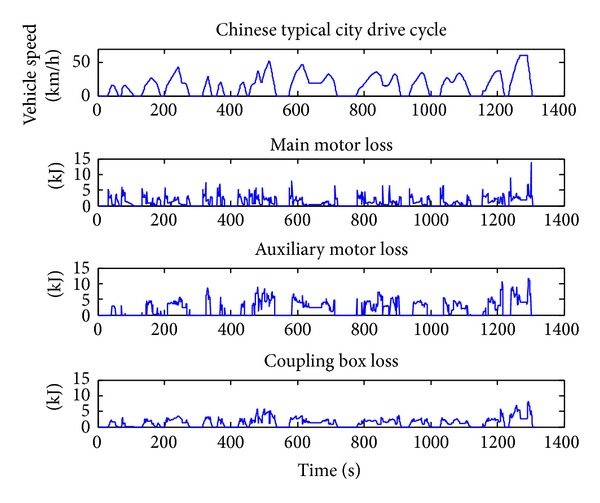
Energy-loss distribution.

When optimizing for only fuel economy, the weighting *α* is set to zero. The Chinese typical city drive cycle is used. The simulation results of the vehicle under the DP policy are shown in Figures 3, 4, and 5. From Figures 3 and 4 we can get that when the vehicle speed is low, the main motor is going to provide the needed speed and power, and when the vehicle speed is high, the motor speed tends to decrease to a very low point and the most vehicle speed and power will be provided by the auxiliary motor. This is because the motor efficiency will be much lower in the working condition of high speed and low output torque. From Figure 2 we can get that the motor efficiency will also be low in the working condition of low speed and low output torque, but in this condition the output power is also low, so the energy loss is lower than that in high speed. Compared with the main motor, the auxiliary motor tends to work on the high speed and high torque condition which is within high efficiency location.

From Figure 5 we can get that the DMCPS's energy loss can be classified into three categories: main motor loss, auxiliary motor loss, and coupling box loss. Among them the auxiliary loss accounts for the main part, while the main motor loss and coupling box loss are almost equal. This is bucause the auxiliary motor is always working in the high power condition although its working efficiency is relatively higher than the main motor.

### 4.2. Energy Loss and Shifting Frequency Optimization Results

**Figure 6:**
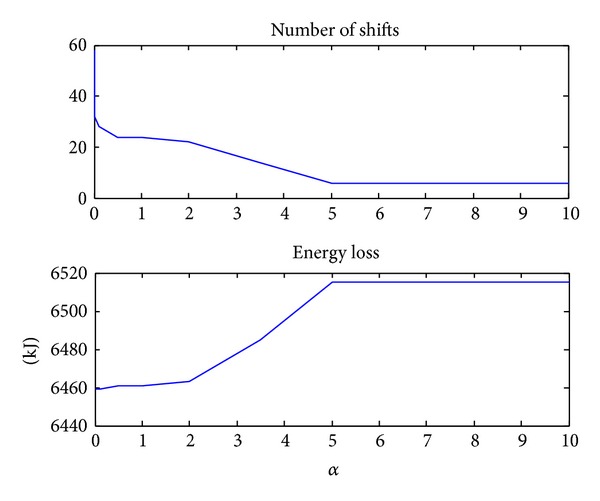
The trend of the energy loss and number of shifts with the change of *α*.

To study the tradeoff between energy loss and shifting frequency the weight factors are varied *α* = [0,0.01,0.1,0.5,1, 2,3.5,5, 10]. The possible values of *α* are chosen based on the reasonable meanings in formula (5). This tradeoff study is important in the early design process because it provides useful information about the sensitivity between the energy loss and shifting frequency. The trend of the energy loss and the number of shifts with the change of *α* are shown in Figure 6. From Figure 6 we can get that when *α* < 1, the number of shifts decreased rapidly (from 58 to 24) with the increase of *α* while the energy loss increased only a little which can be neglected. When *α* increases between 1 and 2, the energy loss and number of shifts only change a little. When *α* increases from 2 to 5, the number of shifts decreases fast again and the energy loss increases fast. When *α* exceeds 5, both the number of shifts and the energy loss stay constant. So the reasonable value will be between 1 and 2. Here we set *α* = 2 for further discussion.

**Figure 7:**
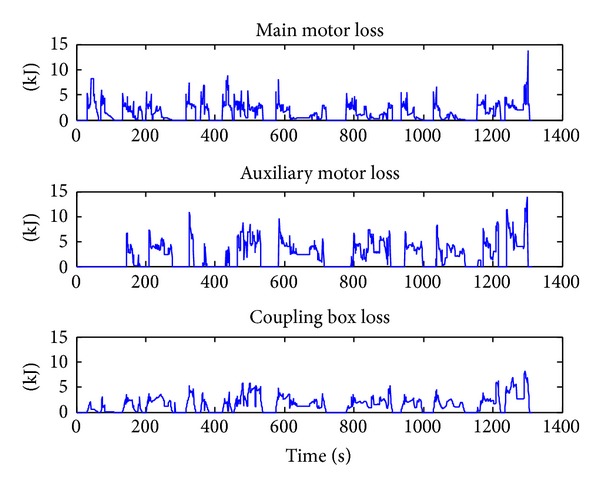
Energy-loss distribution for *α* = 2.

From Figure 7 we can get that to reduce the number of shifts the main motor tends to work more in the low speed. Compared with Figure 5, the number of shifts reduces from 58 to 22, which is only 38% of the original DP results, while the energy loss increased from 5943 to 6431 KJ, which only increased 8.2%.

## 5. Development of Improved Rule-Based Controls

The DP control policy is not implementable in real driving conditions because it requires knowledge of future speed and load profile. Nonetheless, analyzing its behavior provides useful insight into possible improvement of the rule-based controller. Based on the above discussion simulation results, here we abstract the shift control strategy including upshift and downshift strategy and power split strategy.

### 5.1. Working Mode Shift Control

**Figure 8:**
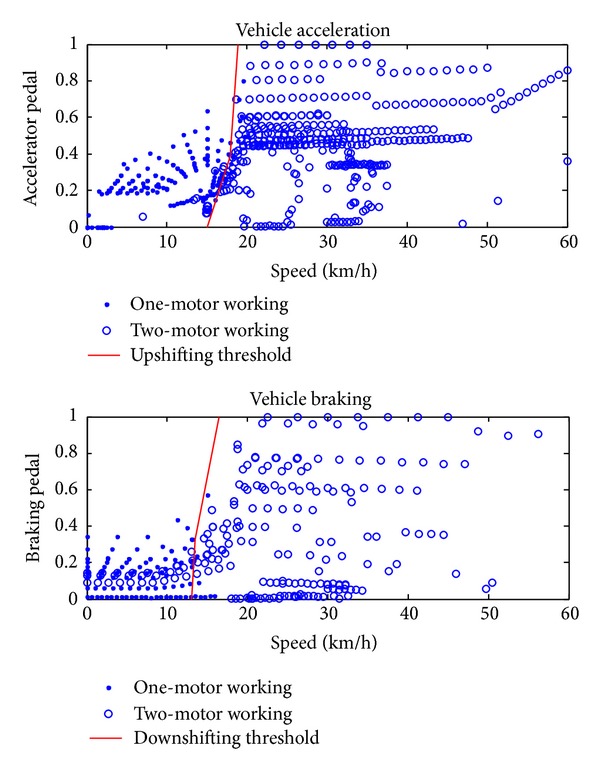
Abstracting of shifting strategy.

**Figure 9:**
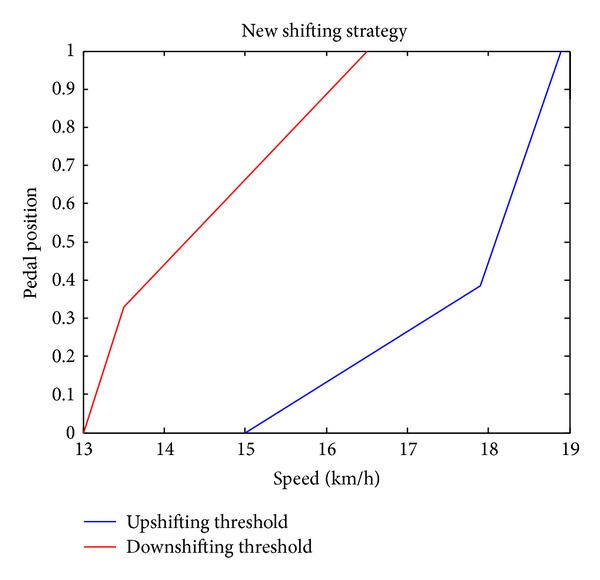
New shifting strategy.

The working mode shift is crucial to the reduction of energy loss and riding comfort. In the original DP results the DMCPS needs frequent shifting to reduce the energy loss, which may influence the riding comfort, and when *α* = 2, the energy loss did not increase a lot but the shifting number is only 38% of the original DP result. Figures 8 and 9 show the abstracting procedure of the downshifting and upshifting threshold based on the DP results data when *α* = 2. In Figure 8 the first graph shows the working condition when the vehicle is accelerating, while the second graph shows the working condition when the vehicle is braking. By drawing a line manually to depart the working mode the shifting strategy can be got. The merit of this work compared with other methods is that this method not only determined the reasonable shifting point but also helped us decide when to upshift and downshift, which can avoid frequent shifting in application. And the result is expressed in Figure 9.

### 5.2. Power Split Control

In this section, we study how power split control of the preliminary rule-based algorithm can be improved by analyzing the DP results when *α* = 2. The power split ratio PR can be expressed as follows:

(16) $$\begin{array}{c} \text{PR}=\frac{P_{r}}{P_{r}+P_{s}}. \end{array}$$

**Figure 10:**
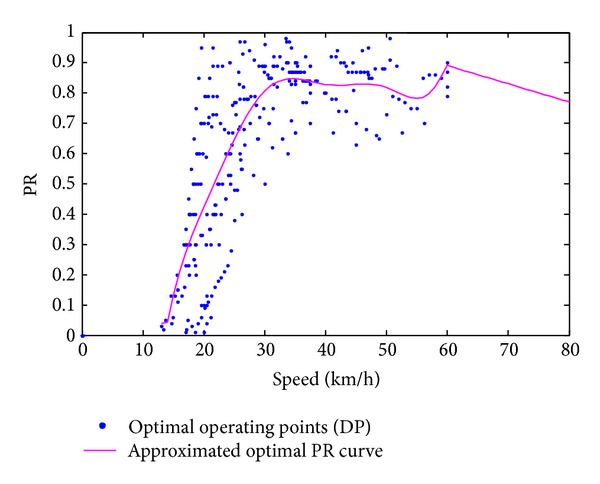
New power split strategy.

Two working modes are defined: single motor working mode (PR = 0) and power coupling working mode (0 < PR < 1). It should be noted that the range of PR is [0,1). In one-motor working mode (PR = 0), as above discussed the control rule is unique. Here we only talk about the coupling condition.  Figure 10 gives the new power split strategy abstracted from the data based on DP results. It can be seen from the curve that the split ratio tends to fluctuate around 0.77 when the speed exceeds 20 km/h; this is because the planet mechanism's property parameter *K* is set to 3.5 and in this ratio the efficiency of coupling box is relatively higher than other ratios. This demonstrates that though the energy loss from coupling box is not the most compared one with the auxiliary motor, it plays an important role in reducing the energy loss.

### 5.3. Performance Evaluation

After incorporating the working mode shift control and power split control outlined in the previous sections, the improved rule-based controller is evaluated using Chinese typical city drive cycle.  Table 2 shows the comparison of different control rules. We can get from the table that the new rule-based strategy can reduce the DMCPS's energy loss effectively. Specificly, the main loss is coming from the main motor in the preliminary rule-based strategy while in the new rule-based strategy and DP operation the main loss is coming from the auxiliary motor. Though the new rule-based strategy reduces the energy loss by about 22%, the DMCPS still has a significant reducing potential as the DP operation reduces the energy loss by 36.9%. From Table 3 we can get that the new rule-based strategy does not need to increase the shifting number but cannot improve the shifting performance too. On the contrary DP (*α* = 2) can realize reducing the shifting number by about 15.34%.

## 6. Conclusion

Based on the simplified model, DP is applied to solve the globally optimal control strategy. Designing the control strategy for DMCPEB by extracting rules from the dynamic programming results has the clear advantage of being near optimal, accommodating multiple objectives, and systematic. Depending on the overall objective, one can easily develop control laws that emphasize low energy loss and riding comfort. By analyzing the DP results the approximate optimal upshift threshold, downshift threshold, and power split ratio were determined. The improved rule-based control strategy can reduce the energy loss by about 22%, while the DP (*α* = 2) can reduce the energy loss by 36.9%.

## Figures and Tables

**Table 1:** Basic parameters of the vehicle and DMCPS.

Name	Value	Unit
Vehicle mass (*M*_vehicle_)	18000	kg
Tire radius (*r*)	0.4785	m
Rolling resistance coefficient (*f*)	0.015	Null
Windward area (*A*)	7.5438	m^2^
Air resistance coefficient (*C*_*D*_)	0.8	Null
Final drive ratio *i*_0_	6.34	Null
Maximum torque of the motor (*T*_max⁡_)	410	Nm
Maximum rotate speed of the motor (*N*_max⁡_)	6000	rpm
Characteristic parameter of PGT (*K*)	3.5	Null

**Table 2:** Comparison of different control strategy in energy loss.

Loss type	Main motor (KJ)	Auxiliary motor (KJ)	Coupling box (KJ)	Total (KJ)	Improvement in total (%)
Preliminary rule-based	5302	2781	2158	10240	0%
New rule-based	2938	3017	2030	7987	22%
DP (*α* = 2)	1848	2691	1922	6461	36.9%

**Table 3:** Comparison of different control strategy in shifting number.

	Shifting number	Improvement in total (%)
Preliminary rule-based	26	0%
New rule-based	26	0%
DP (*α* = 2)	22	15.34%
